# Surveillance for Harmful Algal Bloom Events and Associated Human and Animal Illnesses — One Health Harmful Algal Bloom System, United States, 2016–2018

**DOI:** 10.15585/mmwr.mm6950a2

**Published:** 2020-12-18

**Authors:** Virginia A. Roberts, Marissa Vigar, Lorraine Backer, Gabriella E. Veytsel, Elizabeth D. Hilborn, Elizabeth I. Hamelin, Kayla L. Vanden Esschert, Joana Y. Lively, Jennifer R. Cope, Michele C. Hlavsa, Jonathan S. Yoder

**Affiliations:** ^1^Division of Foodborne, Waterborne, and Environmental Diseases, National Center for Emerging and Zoonotic Infectious Diseases, CDC; ^2^Division of Environmental Health Science and Practice, National Center for Environmental Health, CDC; ^3^Oak Ridge Institute for Science and Education, Oak Ridge, Tennessee; ^4^US Environmental Protection Agency, Research Triangle Park, North Carolina; ^5^Division of Environmental Laboratory Sciences, National Center for Environmental Health, CDC; ^6^Karna, LLC, Atlanta, Georgia.

Harmful algal bloom events can result from the rapid growth, or bloom, of photosynthesizing organisms in natural bodies of fresh, brackish, and salt water. These events can be exacerbated by nutrient pollution (e.g., phosphorus) and warming waters and other climate change effects (*1*); have a negative impact on the health of humans, animals, and the environment; and damage local economies (*2*,*3*). U.S. harmful algal bloom events of public health concern are centered on a subset of phytoplankton: diatoms, dinoflagellates, and cyanobacteria (also called blue-green algae). CDC launched the One Health Harmful Algal Bloom System (OHHABS) in 2016 to inform efforts to prevent human and animal illnesses associated with harmful algal bloom events. A total of 18 states reported 421 harmful algal bloom events, 389 cases of human illness, and 413 cases of animal illness that occurred during 2016–2018. The majority of harmful algal bloom events occurred during May–October (413; 98%) and in freshwater bodies (377; 90%). Human and animal illnesses primarily occurred during June–September (378; 98%) and May–September (410; 100%). Gastrointestinal or generalized illness signs or symptoms were the most frequently reported (>40% of human cases and >50% of animal cases); however, multiple other signs and symptoms were reported. Surveillance data from harmful algal bloom events, exposures, and health effects provide a systematic description of these occurrences and can be used to inform control and prevention of harmful algal bloom–associated illnesses.

Harmful algal bloom events occur in salt, brackish, and fresh water. In bodies of water such as oceans and estuaries, diatoms or dinoflagellates form “tides” that produce toxins associated with seafood poisoning, including paralytic shellfish poisoning, or respiratory distress from inhalation of aerosolized toxins. Cyanobacteria predominantly bloom in fresh water such as lakes and rivers; they can produce microcystins, cylindrospermopsin, and other toxins that humans or animals might be exposed to through water contact, inhalation, or ingestion (*2*,*4*). Animals that become ill or die can be sentinels for harmful algal bloom events. Behavioral and biological factors might increase the likelihood or magnitude of their exposures to toxins compared with human exposures (*5*).

CDC, in consultation with state and federal partners, designed and launched* OHHABS using a One Health^†^ approach. Integrating technical expertise from a 2007–2011 harmful algal bloom surveillance project (*2*) and national waterborne and foodborne outbreak surveillance (https://www.cdc.gov/nors/about.html), the reporting system links harmful algal bloom event data with human or animal illness data and uses standard definitions to classify harmful algal bloom events as suspected or confirmed and cases of human or animal illness as suspected, probable, or confirmed.^§^ Animal illnesses or deaths are reported as single cases or in groups, such as flocks of birds. This summary describes data from OHHABS for January 1, 2016–December 31, 2018, in reports that were submitted^¶^ by state health departments by March 18, 2020. SAS (version 9.4; SAS Institute) was used to conduct descriptive analyses to characterize environmental conditions during harmful algal bloom events, harmful algal bloom–associated cases of human or animal illness, and results of environmental and clinical toxin testing.

A total of 18 states** voluntarily reported 421 harmful algal bloom events that occurred during 2016–2018, with the majority (88%) classified as confirmed (Table 1). These events occurred predominantly during May–October (98%), peaking in July (27%). The majority (90%) of the reported harmful algal bloom events occurred in freshwater bodies. Fewer than one half of all reports (39%) indicated that a visible scum had been observed. Laboratory testing for 372 (88%) harmful algal bloom events was performed on water samples (98%), algae or cyanobacteria (7%), or food samples (1%). Reasons for testing included environmental monitoring activities (79%), citizen complaints (17%), or health events involving animals (5%) or humans (5%). Toxin results reported for 308 harmful algal bloom events (83%) frequently identified microcystins (94%); 35 (11%) reports identified more than one type of toxin.

**TABLE 1:** Characteristics of harmful algal bloom events (n = 421) — One Health Harmful Algal Bloom System (OHHABS),* United States, 2016–2018

Characteristic	No. (%)
Classification^†^	
Confirmed	370 (88)
Suspected	51 (12)
Water source type	
Fresh	377 (90)
Brackish	14 (3)
Salt	12 (3)
Unknown	18 (4)
Month^§,¶^	
February	1 (—)
March	2 (—)
April	3 (1)
May	31 (7)
June	65 (15)
July	115 (27)
August	99 (24)
September	75 (18)
October	28 (7)
Unknown	2 (—)
Scum observed	166 (39)
Laboratory testing performed	372 (88)
Sample type tested**	
Raw or ambient water	363 (98)
Algae or cyanobacteria	27 (7)
Food	4 (1)
Finished drinking water	1 (—)
Reason for testing**	
Monitoring	295 (79)
Citizen complaint	64 (17)
Animal health event response	17 (5)
Human health event response	17 (5)
Fish illness or kill	4 (1)
Other	3 (1)
Odor	1 (—)
Unknown	7 (2)
Testing results**^,††^	
Toxins^§§^	308 (83)
Microcystins	291 (94)
Anatoxin-A	30 (10)
Saxitoxin	19 (6)
Cylindrospermopsin	4 (1)
Nodularin	3 (1)
Ciguatoxin	1 (—)
Other	1 (—)
Cyanobacteria	65 (17)
Dinoflagellates	8 (2)
Gonyaulacales	2 (25)
Gymnodiniales	3 (38)
Peridiniales	1 (13)
Prorocentrales	1 (13)
Unknown	2 (25)
**Raphidophyceans**	**3 (1)**
**Diatoms**	**2 (1)**
**Unknown^¶¶^**	**23 (7)**

* A total of 18 states adopted OHHABS and voluntarily reported 421 harmful algal bloom events: Alaska, Arizona, California, Connecticut, Florida, Kansas, Maryland, Michigan, Minnesota, Nevada, New York, North Carolina, Ohio, Oregon, Pennsylvania, Utah, Virginia, and Wisconsin.

^†^ Event classification criteria are located at https://www.cdc.gov/habs/pdf/ohhabs-case-and-event-definitions-table-508.pdf.

^§^ Percentages do not sum to 100% as a result of rounding.

^¶^ Month was assigned based on data availability, using the following hierarchy: 1) bloom observation date, 2) month of bloom notification, and 3) earliest date of an associated human or animal case.

** Percentages might exceed 100% because multiple options could be selected.

^††^ Data collection was restricted to positive results from environmental testing.

^§§^ Multiple toxins were reported for 35 events, with microcystins as one of the toxin classifications detected in 33 events. Other toxins detected included anatoxin-a, cylindrospermopsin, nodularin, saxitoxin, or other unspecified toxins.

^¶¶^ Twenty-three reports did not include results from environmental testing.

A total of 389 human cases of illness were reported, with 341 (88%) classified as probable (Table 2). Approximately one half of cases (199; 51%) resulted from a single, freshwater harmful algal bloom event that occurred in a large lake in July; extended to connected waterways, such as rivers, canals, and reservoirs; and spanned more than 3 months. At least 153 (39%) of the 389 persons with cases were aged <18 years. Almost all (98%) reported illnesses occurred during June–September. Signs and symptoms reported for 380 (98%) cases indicated that affected persons most frequently experienced gastrointestinal (67%); generalized (e.g., headache, fever, or lethargy) (43%); dermatologic (27%); or ear, nose, or throat-related (16%) signs or symptoms. No deaths were reported. Time to onset of initial signs or symptoms was available for 124 persons who had a one-time exposure and ranged from 1 minute to 8 days. Patients consulted poison control centers (76%), health care providers (17%), or emergency departments (9%). Clinical specimens were tested for 30 (8%) patients; CDC performed urinalysis for five of these persons and confirmed that four had exposures to saxitoxin or multiple toxins.

**TABLE 2:** Characteristics of human exposure and illness (n = 389*) associated with harmful algal bloom events (n = 73) — One Health Harmful Algal Bloom System (OHHABS),^†^ 2016–2018

Human case characteristic	No. (%)
Classification^§^	
Confirmed	14 (4)
Probable	341 (87)
Suspected	34 (9)
Water source type^¶^	
Fresh	366 (94)
Brackish	1 (—)
Salt	0 (—)
Unknown	22 (6)
Age group, yrs**	
0–1	3 (1)
2–4	39 (10)
5–11	50 (13)
12–17	61 (16)
18–45	137 (35)
46–64	42 (11)
≥65	18 (5)
Unknown	39 (10)
Sex**	
Male	200 (51)
Female	184 (47)
Unknown	5 (1)
Month of illness onset	
February	1 (—)
May	5 (1)
June	34 (9)
July	260 (67)
August	57 (15)
September	27 (7)
October	4 (1)
Unknown	1 (—)
Setting of exposure^††,§§^	
Public outdoor area	235 (60)
Beach	63 (16)
Private residence	53 (14)
Other	57 (15)
Park	27 (7)
Unknown	22 (6)
Health care seeking behavior^††^	
Call to a poison control center	297 (76)
Health care provider	68 (17)
Emergency department	36 (9)
First aid care	3 (1)
Clinical testing^¶¶^	30 (8)
Signs and symptoms^††,^***^,†††^	
Gastrointestinal	262 (67)
Generalized	169 (43)
Dermatologic	104 (27)
Ear, nose, or throat	62 (16)
Neurologic	56 (14)
Cardiopulmonary	41 (11)
Ophthalmologic	30 (8)
Other	30 (8)
Musculoskeletal	13 (3)
Genitourinary	6 (2)
Unknown	9 (2)
Median time to illness onset—one-time exposure, hours (min, max) (n = 124 cases)	13.5 (0.02–192)
Foodborne illness**	22 (6)
Paralytic shellfish poisoning (PSP)	11 (50)
Ciguatera fish poisoning (CFP)	5 (23)
Other	4 (18)
Unknown	2 (10)

* A total of 199 (51%) cases were the result of a single freshwater harmful algal bloom event.

^†^ Of 18 states that adopted OHHABS, 10 states voluntarily reported 389 cases of human illness: Alaska, California, Kansas, Minnesota, New York, Ohio, Oregon, Pennsylvania, Utah, and Wisconsin.

^§^ Case classification criteria are available at https://www.cdc.gov/habs/pdf/ohhabs-case-and-event-definitions-table-508.pdf.

^¶^ Water source type is the water body type from the linked harmful algal bloom event.

** Percentages do not sum to 100% as a result of rounding.

^††^ Percentages might exceed 100% because multiple options could be selected.

^§§^ Other setting category includes ship, outdoor place of work, camp or cabin setting, farm or agricultural setting, resort, National Forest, school, college, or university, subdivision or neighborhood, apartment or condo, hotel, motel, lodge, or inn, and other unspecified settings.

^¶¶^ Specimens for five cases of foodborne illness were tested at CDC; urinalysis confirmed exposures to saxitoxin or multiple toxins for four of five patients.

*** Signs and symptoms were classified primarily based on the biological system that was affected. "Generalized" refers to constitutional signs and symptoms such as headache, fever, or lethargy. Some signs and symptoms that have been classified as neurologic might present in other systems (e.g., ophthalmologic). Classifications are available at https://www.cdc.gov/habs/ohhabs_tables_and_figures.html.

^†††^ Sixty-seven percent of persons with cases were still experiencing symptoms at the time of interview.

Based on 64 animal case reports, at least 413 animals^††^ became ill, and 369 (89%) died (Table 3). The majority (81%) of animal cases of illness were classified as suspected. The majority (89%) of the exposures involved fresh water, including one large bird die-off of 300 (73%) cases that occurred at a lake in May 2018. Almost all (99%) illnesses^§§^ occurred during May–September. Within animal categories of domestic pets (52), livestock (42), and wildlife (319), the most frequently affected animals were dogs (96%), cattle (86%), and birds (97%). Signs of illness were available for 92 cases and included generalized (e.g., weakness, lethargy, or anorexia) (64%), gastrointestinal (54%), and neurologic (14%) symptoms. Time to onset of initial signs was available for 21 animals that had a one-time exposure and ranged from 15 minutes to 4 days. Veterinary medical care or treatment was provided to 25 (6%) animals.

**TABLE 3:** Characteristics of animal exposure and illness (n = 413) associated with harmful algal bloom events (n = 42) — One Health Harmful Algal Bloom System (OHHABS),* 2016–2018

Animal case characteristic	No. (%)
Single or group case report (n = 64)^†^	
Single	55 (86)
Group^§^	9 (14)
Deaths	369 (89)
Classification^¶^	
Confirmed	13 (3)
Probable	67 (16)
Suspected	333 (81)
Water source type**	
Fresh	366 (89)
Brackish	11 (3)
Salt	2 (—)
Unknown	34 (8)
Category	
Domestic pet	52 (13)
Dog	50 (96)
Cat	2 (4)
Livestock^††^	42 (10)
Cattle	36 (86)
Bird	4 (10)
Horse or donkey	2 (5)
Wildlife^††^	319 (77)
Bird	310 (97)
Fish	6 (2)
Other mammal	1 (—)
Month of illness^§§^	
May	304 (74)
June	36 (9)
July	28 (7)
August	32 (8)
September	10 (2)
October	1 (—)
December	2 (—)
Setting of exposure^¶¶,^***^,§§§^	112 (27)
Private residence	29 (26)
Public outdoor area	22 (20)
Other	18 (16)
Beach	13 (12)
Unknown	37 (33)
Veterinary medical care or treatment received^†††^	25 (6)
Signs ^¶¶,§§§,¶¶¶^	92 (22)
Generalized	59 (64)
Gastrointestinal	50 (54)
Neurologic	13 (14)
Cardiopulmonary	7 (8)
Ophthalmologic	5 (5)
Other	3 (3)
Dermatologic	2 (2)
Ear, nose, or throat	1 (1)
Hematologic	1 (1)
Median time to illness onset – one-time exposure, hours (min, max) (n = 21)	6 (0.25–96)

* Of 18 states that adopted OHHABS, 10 states voluntarily reported 413 cases of animal illness: California, Florida, Kansas, Michigan, Minnesota, New York, Oregon, Utah, Virginia, and Wisconsin.

^†^ Animals could be reported as single cases or in groups on a single form. If no total case count was reported for a group of animals, a value of two animal cases was assigned. Data provided in aggregate for groups of animals were extrapolated to describe exposures, case attributes, and health effects.

^§^ Birds (three), cattle (two), fish (two), dogs (one), and horses (one). These groups included a large bird die-off (300).

^¶^ Case classification criteria are available at https://www.cdc.gov/habs/pdf/ohhabs-case-and-event-definitions-table-508.pdf.

** Water source is the water body type from the linked harmful algal bloom event.

^††^ Percentages do not sum to 100% as a result of rounding.

^§§^ Animal cases were assigned months based on data availability, using the following hierarchy: 1) illness onset date, 2) discovery date, and 3) death date.

^¶¶^ Summarized for the subset of reports with data available.

*** Other setting category includes park, community or municipality, resort, or ship.

^†††^ Animal group reports were manually reviewed. If multiple animals were reported as receiving care but no total case count could be confirmed, a value of two animal cases was assigned.

^§§§^ Percentages might exceed 100% because multiple options could be selected.

^¶¶¶^ Signs were available for 49 dogs, 36 cattle, four birds, two horses and one cat. Signs were classified primarily based on the biological system that was affected. "Generalized" refers to constitutional signs such as weakness, lethargy, or anorexia. Some signs that have been classified as neurologic might present in other system (e.g., ophthalmologic). Classifications are available at https://www.cdc.gov/habs/ohhabs_tables_and_figures.html.

## Discussion

Data reported to OHHABS by 18 states for 2016–2018 included 421 harmful algal bloom events, 389 cases of human illness, and 413 cases of animal illness. While the majority of harmful algal bloom events were classified as confirmed, the majority of human illnesses were classified as probable, and animal illnesses as suspected. These data reflect the launch of national public health surveillance for harmful algal bloom events and associated illnesses in the United States.

Epidemiologists, environmental health practitioners, public health laboratorians, and health communicators work together to increase awareness and understanding of public health risks of harmful algal bloom events. In addition, animal health professionals, environmental health professionals, and other stakeholders, such as academics, parks and recreation professionals, and citizen scientists, have knowledge and networks that strengthen the public health system’s ability to detect, investigate, and report harmful algal bloom events and associated illnesses (*2*,*6*). Poison control centers also can support case detection and investigation by sharing data with health departments. Illustrative of this approach, the Utah Poison Control Center shared data with the Utah Department of Health and entered data into OHHABS, including during a cyanobacterial bloom event in 2016 that resulted in half of the total human cases reported for 2016–2018.

Diagnostic testing for harmful algal bloom toxins is under development but is not currently available in routine clinical settings (*7*). Fewer than 5% of human or animal cases of illness were classified as confirmed on the basis of current OHHABS criteria, which require supporting evidence such as a medical diagnosis or clinical confirmation of a harmful algal bloom exposure. Health care providers can play a critical role by notifying health departments when they suspect harmful algal bloom–associated illnesses, considering harmful algal bloom–associated illness as a differential diagnosis, and assigning relevant *International Classification of Diseases* (ICD) codes (*8*). More access to confirmatory testing is needed to support public health surveillance.

Laboratory testing occurred in approximately 90% of harmful algal bloom events, most often related to environmental monitoring activities, citizen complaints, or health event responses. Many U.S. jurisdictions have developed programs to monitor harmful algal blooms and related tools that help communicate health risks from exposure to harmful algal bloom events.^¶¶^ Remote sensing (e.g., satellite imagery) and citizen scientist opportunities can supplement such efforts and might help to increase early detection of harmful algal bloom events.***^,†††,§§§^ Recently, the U.S. Environmental Protection Agency released risk-based guidance for microcystins and cylindrospermopsin to assist with management of drinking water systems and recreational bodies of water (*9*,*10*). This guidance, paired with water monitoring activities, notification systems, and community engagement, might be used to increase the completeness and accuracy of public health surveillance data reported in OHHABS, thereby increasing the data available for public health decision-making.

The findings in this report are subject to at least four limitations. First, these data are for the initial OHHABS data collection period; participation, data completeness, and data quality are anticipated to improve over time. Second, the number of reported events or illnesses underrepresents the total that occurred within or across states. Surveillance capacity and scope (e.g., inclusion of animal case reports) vary across jurisdictions and within this reporting period. Third, case and event definitions are not toxin-specific and do not yet have thresholds for test results from clinical specimen or environmental samples that correspond to acute health outcomes or public health action levels for toxins. Finally, harmful algal bloom events can exhibit geospatial, temporal, and toxin production variability, which makes environmental data more difficult to collect, interpret, and report.

OHHABS is informed by local, state, and federal One Health partnerships. Data about harmful algal bloom exposures and health effects can be used to support prevention of harmful algal bloom–associated illnesses, which might increase because of warming waters or other climate change impacts (https://www.epa.gov/nutrientpollution/climate-change-and-harmful-algal-blooms). OHHABS data can inform educational resources and outreach efforts by identifying factors that contribute to illnesses and informing targeted messages to populations at risk. More in-depth analyses to further characterize the data should support additional public health policy and prevention efforts. A continued One Health approach to surveillance, paired with scientific research (e.g., environmental science and human and animal health studies) findings and increased access to specimen testing, will add to the robustness and utility of the system.

**Summary**

**What is already known on this topic?**

Harmful algal blooms occur in fresh, brackish, and salt water throughout the United States. They can affect human and animal health and have ecological and economic impacts.

**What is added by this report?**

Eighteen states adopted use of the One Health Harmful Algal Bloom System and entered 421 reports during 2016–2018, including information about 389 human illnesses and at least 413 animal illnesses associated with harmful algal bloom events.

**What are the implications for public health practice?**

Information about harmful algal bloom exposures and health effects support efforts to detect these events and mitigate and prevent associated illnesses. Human, animal, and environmental health partners can work together to document the occurrence and impacts of harmful algal bloom events and characterize associated illnesses.

## Footnotes

* OHHABS partners and system development are described in more detail at https://www.cdc.gov/habs/ohhabs_tables_and_figures.html.

† One Health is defined by CDC as “a collaborative, multisectoral, and transdisciplinary approach — working at the local, regional, national, and global levels — with the goal of achieving optimal health outcomes recognizing the interconnection between persons, animals, plants, and their shared environment.” https://www.cdc.gov/onehealth/index.html.

§ Harmful algal bloom events and cases reported to OHHABS are classified based on standard definitions. Suspected harmful algal bloom events are required to have observational/environmental data or associated illnesses as supporting evidence. Confirmed harmful algal bloom events are required to have either laboratory-based harmful algal bloom data or both observational/environmental data and at least one associated case of illness as supporting evidence. Harmful algal bloom–associated illness data are subject to a public health assessment process. Suspected human or animal cases must have experienced a harmful algal bloom exposure and signs/symptoms. Probable human or animal case classifications must be supported by one of the following: observational/environmental data, laboratory-based harmful algal bloom data, or a professional medical diagnosis. Confirmed human or animal cases must be supported by one of the following: 1) clinical data confirming the exposure plus a professional medical diagnosis or other causes ruled out or 2) laboratory-based harmful algal bloom data plus a professional medical diagnosis and other causes ruled out. More detailed information is available at https://www.cdc.gov/habs/pdf/ohhabs-case-and-event-definitions-table-508.pdf.

¶ OHHABS is available for voluntary reporting by public health agencies and their designated environmental health or animal health partners in the United States, District of Columbia, Federated States of Micronesia, Guam, Marshall Islands, Northern Mariana Islands, Palau, Puerto Rico, and U.S. Virgin Islands.

** Alaska, Arizona, California, Connecticut, Florida, Kansas, Maryland, Michigan, Minnesota, Nevada, New York, North Carolina, Ohio, Oregon, Pennsylvania, Utah, Virginia, and Wisconsin.

†† If no total case count was reported for a group of animals, data were extrapolated, with a value of two animal cases assigned as a conservative estimate.

§§ Animal cases were assigned months based on data availability, using the following hierarchy: 1) illness onset date, 2) discovery date, and 3) death date.

¶¶ https://www.epa.gov/cyanohabs/state-habs-monitoring-programs-and-resources.

*** https://oceanservice.noaa.gov/hazards/hab.
